# Multi-Omics Analysis Reveals Molecular Networks and Key Pathways Associated with Cysteine- and Methionine-Mediated Biosynthesis of Sulfur-Containing Flavor Metabolites in *Lentinula edodes*

**DOI:** 10.3390/ijms27177744

**Published:** 2026-08-29

**Authors:** Yuan Guo, Yu Chen, Yajie Zhang, Shuang Song, Dianpeng Zhang, Caige Lu, Qi Gao, Yu Liu, Shouxian Wang

**Affiliations:** Institute of Plant Protection, Beijing Engineering Research Center for Edible Mushroom, Beijing Academy of Agriculture and Forestry Sciences, Beijing 100097, China; guoyuan@baafs.net.cn (Y.G.);

**Keywords:** *Lentinula edodes*, sulfur-containing flavor metabolites, proteomics, metabolomics, omics integration, metabolic pathway

## Abstract

*Lentinula edodes* is renowned for its unique aroma, which is characterized by various volatile sulfur-containing flavor metabolites (SCFMs). Cysteine and methionine could enhance the SCFMs biosynthesis in *L. edodes*; however, the underlying metabolic pathways remain unclear. To bridge this gap, integrated proteomic and metabolomic analysis were performed to decipher pathways through which cysteine and methionine regulate SCFM biosynthesis. Results showed that exogenous cysteine and methionine supplementation significantly increased the content of lenthionine, the key aroma compound of shiitake mushrooms. Both treatments induced substantial changes in the proteomic and metabolomic profiles. Proteomic analysis revealed that differentially expressed proteins were predominantly enriched in cysteine and methionine metabolism and sulfur metabolism following cysteine treatment, whereas methionine treatment mainly affected proteins associated with tryptophan metabolism and sulfur metabolism. Metabolomic analysis showed that differentially accumulated metabolites were significantly enriched in D-amino acid metabolism and cysteine and methionine metabolism, with glutathione metabolism specifically enriched under cysteine treatment. Integrated omics analysis further uncovered distinct sulfur metabolite–protein regulatory networks under different sulfur nutrition and identified treatment-specific hub proteins. These findings establish a molecular regulatory framework linking SCFM biosynthesis with broader primary metabolic pathways involved in sulfur intermediate generation and regulation, providing new insights into the potential regulatory networks underlying SCFM formation in *L. edodes*.

## 1. Introduction

*Lentinula edodes*, commonly known as xianggu or shiitake, is the second-most widely cultivated and consumed edible mushroom worldwide [1,2]. Its characteristic aroma is one of the key quality attributes that drives consumer preference and underpins its commercial value [3,4]. Its unique aroma is considered an important indicator of the sensory quality and commercial value of *L. edodes* fruiting bodies [3,5,6,7].

The characteristic aroma of *L. edodes* is primarily attributed to a diverse array of volatile sulfur-containing flavor metabolites (SCFMs) [5,8,9]. These include sulfur-containing heterocycles, such as 1,2,4-trithiolane, lenthionine (1,2,3,5,6-pentathiepane), 1,2,4,6-tetrathiepane, and 1,2,4,5-tetrathiane, as well as low-molecular-weight acyclic sulfur compounds, including dimethyl disulfide, carbon disulfide, hydrogen sulfide, and dimethyl trisulfide, etc. [10,11,12]. The SCFMs account for approximately 19.41–21.53% of the total volatile compounds in fresh shiitake fruiting bodies and 22.91–74.74% in dried shiitake mushrooms [12]. In addition to fruiting bodies, the SCFMs are also present in mycelia of *L. edodes*, and their composition and abundance vary across different developmental stages [13].

The biosynthesis of SCFMs in *L. edodes* involves two enzymatic reactions followed by a non-enzymatic reaction. First, the γ-glutamyl transpeptidase (GGT) catalyzes the conversion of lentinic acid to γ-glutamyl-lentinic acid by removing the γ-glutamyl peptide bond. Subsequently, cysteine sulfoxide lyase (CSL or C–S lyase) converts γ-glutamyl-lentinic acid into thiosulfinate intermediates. These intermediates are then converted into various sulfur-containing flavor compounds through a series of non-enzymatic condensation and cleavage reactions [14,15,16]. Except for these known pathways, the biosynthetic origin of lentinic acid remains unclear. Moreover, understanding of the relationship between SCFMs and the primary sulfur metabolic pathways in *L. edodes* is still in its infancy. Some of the metabolic pathways, such as sulfur metabolism (KEGG pathway: ko00920), cysteine and methionine metabolism (ko00270), glutathione metabolism (ko00480), and amino acid metabolism (ko00470) are known to provide versatile sulfur-containing intermediates and precursors that may contribute to sulfur-flavor formation. How SCFM formation is coordinated with these known metabolic pathways, however, remains vague.

Formation of the characteristic aroma of *L. edodes* is influenced by the sulfur content of the growth substrate [5,17]. Previous studies have demonstrated that supplementation with sulfur-containing amino acids, including methionine and cysteine, as well as inorganic sulfur sources such as ammonium sulfate and sodium sulfate, significantly promote the biosynthesis of 1,2,4-trithiolane, an important indicator of aroma intensity in dried shiitake mushrooms [17]. Among these sulfur sources, cysteine exhibited the most pronounced effect, followed by methionine [3,17]. However, the interpretation of these findings could be limited because neither lenthionine, the principal sulfur-containing aroma compound in shiitake mushrooms, nor the total sulfur-containing volatile profile was investigated. In addition, several low-molecular-weight sulfur-containing flavor compounds, including dimethyl sulfide, dimethyl disulfide, and dimethyl trisulfide, have been reported to originate from the sulfur-containing amino acids methionine and cysteine [18]. In addition to the normal growth conditions, cysteine regulates lenthionine biosynthesis under drought stress conditions [15]. Although previous findings suggest the involvement of cysteine and methionine in the formation of SCFMs in *L. edodes*, direct experimental evidence defining the specific anabolic pathways and molecular regulatory mechanisms remains limited.

In this study, the effects of cysteine and methionine on the production of lenthionine were investigated in mycelia of *L. edodes*. Integrated proteomic and metabolomic analyses were further employed to elucidate the metabolic pathways and identify candidate functional proteins involved in cysteine- and methionine-mediated regulation of SCFM biosynthesis.

## 2. Results

### 2.1. Influences of Exogenous Cysteine and Methionine Supplementation on Mycelial Growth of L. edodes

**Figure 1:**
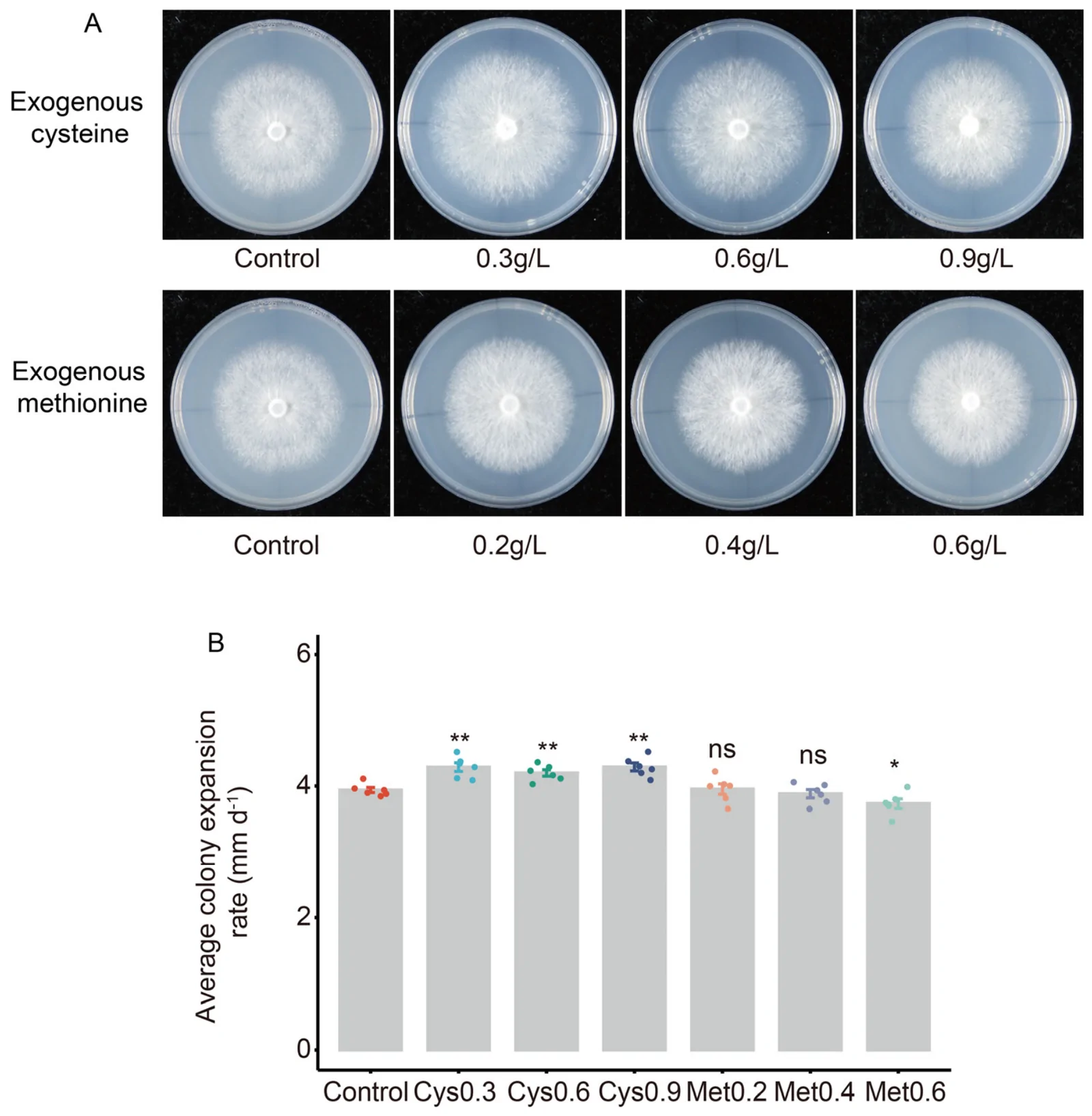
Influences of cysteine and methionine additions on phenotypes (**A**) and average colony expansion rates (**B**) of *L. edodes* mycelia. Data are presented as mean ± SEM (*n* = 6 independent biological replicates). Error bars represent the standard error of the mean (SEM). * and ** indicate significant differences at *p* < 0.05 and <0.01, respectively; ns indicates no significant difference.

To more precisely examine the effects of exogenous cysteine and methionine, a cysteine- and methionine-free media was used. Other sulfur sources, including MgSO_4_·7H_2_O and thiamine hydrochloride, were retained in the medium to support the vegetative growth of *L. edodes*. These two sulfur compounds were maintained at identical concentrations among all experimental groups to eliminate the background effects. Based on the morphological traits and growth of fungal colonies, the modified medium was considered supportive of normal vegetative growth of *L. edodes* (Figure 1A). Compared with the control, the most pronounced changes in mycelial growth were observed following supplementation with 0.6 g/L cysteine (Cys) and 0.2 g/L methionine (Met). No visible morphological differences could be identified in response to exogenous cysteine and methionine (Figure 1A). All tested concentrations of cysteine (0.3–0.9 g/L) significantly enhanced mycelial growth. In contrast, methionine supplementation at 0.2 g/L and 0.4 g/L did not significantly affect average colony expansion rate, whereas 0.6 g/L methionine markedly inhibited mycelial growth (Figure 1B).

### 2.2. Exogenous Cysteine and Methionine Enhanced Lenthionine Synthesis

**Figure 2:**
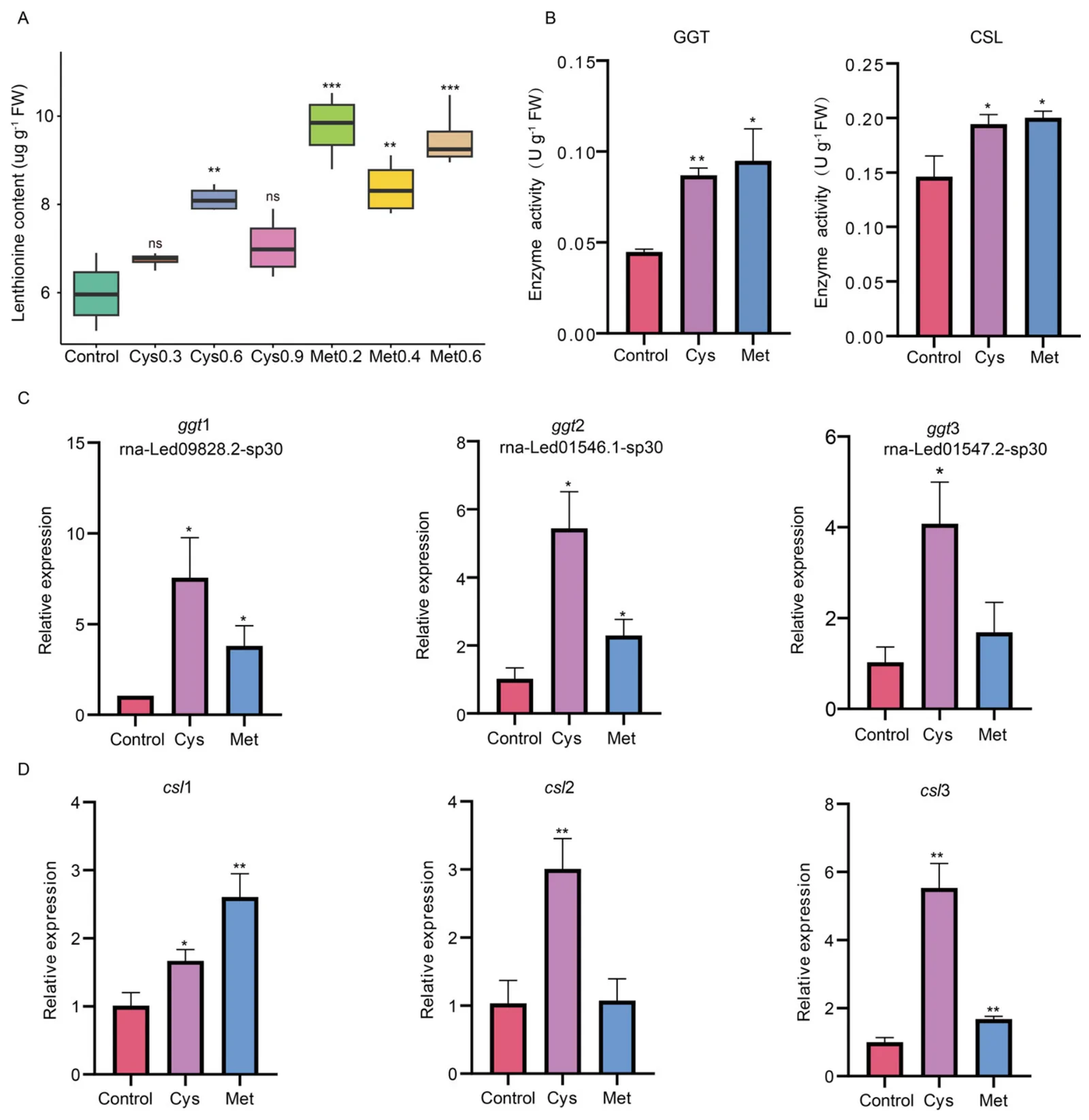
Effects of exogenous cysteine and methionine on lenthionine synthesis. (**A**) Lenthionine content in response to different concentrations of Cys and Met. (**B**) Activities of γ-glutamyl transpeptidase (GGT) and cysteine sulfoxide lyase (CSL). (**C**) Relative expression levels of *ggt* genes. (**D**) Relative expression levels of *csl* genes. Data are presented as mean ± SEM (*n* = 3 biological replicates), and each data point represents the mean value of 3 technical replicates. Error bars represent the standard error of the mean (SEM). *, ** and *** indicate significant differences at *p* < 0.05, <0.01 and <0.001, respectively; “ns” indicates no significant difference. FW denotes fresh weight.

Compared with the control group, exogenous cysteine supplementation significantly increased the endogenous cysteine content in *L. edodes* mycelia, suggesting that additional cysteine supply enhanced intracellular cysteine accumulation and provided increased sulfur precursor availability for subsequent lenthionine biosynthesis (Supplementary Figure S1). Lenthionine, the key metabolite representing shiitake’s characteristic flavor, was significantly elevated by additions of 0.6 g/L cysteine (Cys) and 0.2–0.6 g/L methionine (Met), with 0.2 g/L Met causing the strongest effect (Figure 2A). Consequently, 0.6 g/L Cys and 0.2 g/L Met were selected to assess effects on activities of γ-glutamyl transpeptidase (GGT) and cysteine sulfoxide lyase (CSL), the key enzymes regulating sulfur-flavor biosynthesis. Both treatments significantly increased the activities of GGT and CSL (Figure 2B).

qPCR analysis revealed that Cys and Met markedly upregulated three *ggt* genes (*ggt1*–*3*), with cysteine exerting the greatest effect (Figure 2C). Similarly, all three *csl* genes (*csl1–3*) were induced; Met had the strongest effect on *csl1*, while Cys predominated for *csl2* and *csl3* (Figure 2D). These results indicate that exogenous Cys and Met differentially regulate enzyme activities and gene expression to enhance lenthionine production.

### 2.3. Exogenous Cys and Met Altered the Proteomic Profiles of L. edodes Mycelium

**Figure 3:**
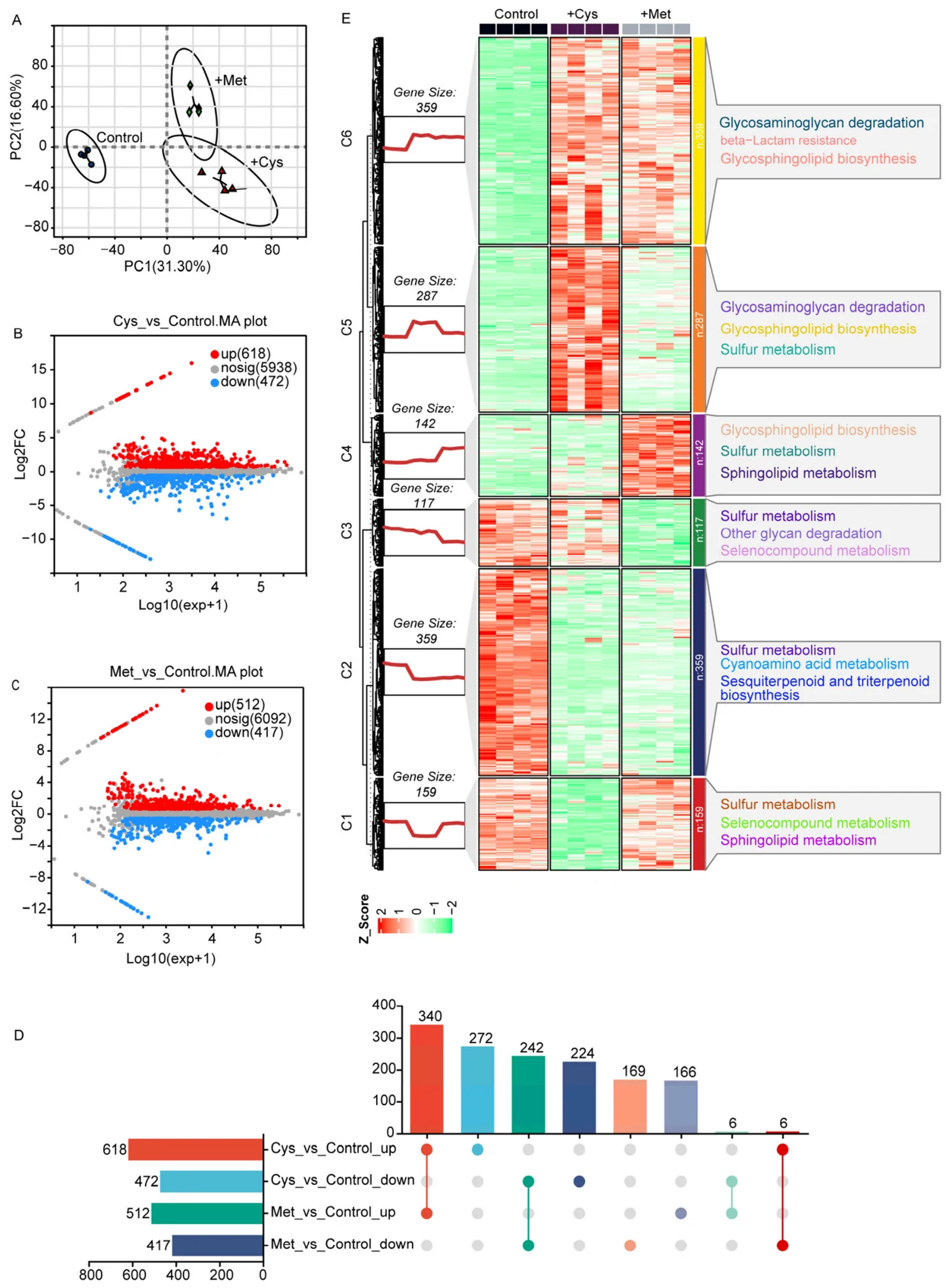
Effects of exogenous cysteine (Cys) and methionine (Met) on the proteomic profiles of *L. edodes* mycelium. (**A**) Principal component analysis (PCA) of proteomic datasets. (**B**,**C**) Volcano plots showing differentially expressed proteins (DEPs) in response to Cys (**B**) and Met (**C**) supplementation. (**D**) UpSet plot showing shared and treatment-specific DEPs between the Cys and Met groups. (**E**) Hierarchical clustering heatmap of representative DEPs in the control and treatment groups.

Non-targeted proteomic analysis identified a total of 7205 proteins, of which 81.42% were annotated in the Pfam database (Supplementary Table S1). The annotations and expression levels of all identified proteins are provided in Supplementary Table S2. PCA revealed distinct proteomic profiles of mycelium in response to Cys and Met supplementation. The first principal component (PC1) accounted for 31.3% of the total variance, separating samples with and without exogenous Cys and Met. The second principal component (PC2), explaining 16.6% of the variance, distinguished between Cys- and Met-treated samples (Figure 3A). Volcano plot analysis (Figure 3B,C) identified 1090 differentially expressed proteins (DEPs) between the control and Cys groups (618 upregulated, 472 downregulated) and 929 DEPs between the control and Met groups (512 upregulated, 417 downregulated). The UpSet plot showed that 340 DEPs were commonly upregulated in both Cys and Met treatments, while 242 DEPs were commonly downregulated. These shared DEPs may serve as potential biomarkers and highlight conserved protein expression responses to the two exogenous8 additives (Figure 3D).

Heatmap analysis clustered these proteins into six groups (C1–C6). Clusters C2 and C3, primarily associated with sulfur metabolism, were more highly expressed in the control group, whereas clusters C4–C6 exhibited higher expression in response to Cys and Met supplementation (Figure 3E).

### 2.4. KEGG Pathway Enrichment Analysis Revealed Different Pathways in Response to Cys and Met Supplement

**Figure 4:**
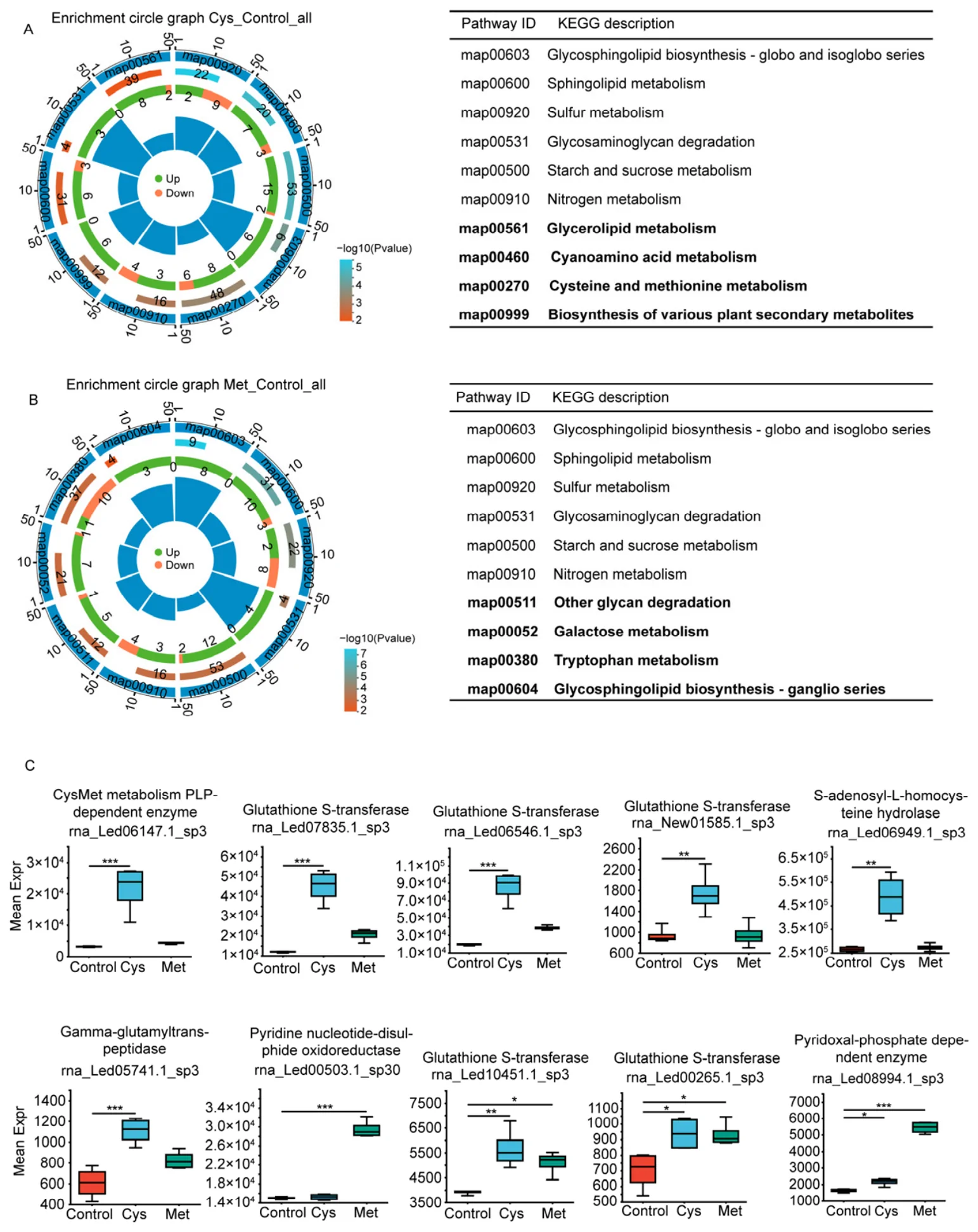
KEGG pathway enrichment analysis of differentially expressed proteins in response to cysteine (**A**) and methionine (**B**). The enrichment circle plot consists of four concentric circles from the outside to the inside. The first circle represents the enriched functional categories, with different colors indicating distinct categories. The second circle shows the number of background proteins in each category and the corresponding *p* value; longer bars correspond to a greater number of proteins, and a smaller *p* value is represented by a darker blue color, indicating more significant enrichment. The third circle is a bar chart illustrating the proportion of upregulated and downregulated proteins, with specific values labeled below. The fourth circle displays the Rich Factor value for each category. (**C**) Boxplot of selected proteins. *, ** and *** indicate significant differences at *p* < 0.05, <0.01 and <0.001, respectively.

KEGG pathway enrichment analysis was performed using treatment-specific DEPs. Among the top 10 significantly enriched pathways, five were shared between the exogenous Cys and Met treatments. Exogenous Cys specifically enriched DEPs associated with glycerolipid metabolism, cysteine and methionine metabolism, biosynthesis of various secondary metabolites in plants, and cyanoamino acid metabolism (Figure 4A). In contrast, exogenous Met specifically enriched DEPs involved in sphingolipid biosynthesis, degradation of other glycans, tryptophan metabolism, and galactose metabolism (Figure 4B).

DEPs associated with sulfur-containing metabolite metabolism are shown in Figure 4C. Five glutathione S-transferases were upregulated in response to exogenous Cys, whereas only two were upregulated following Met treatment. The CysMet metabolism PLP-dependent enzyme, S-adenosyl-L-homocysteine hydrolase, and Gamma-glutamyltranspeptidase were exclusively upregulated by exogenous Cys. In contrast, pyridine nucleotide-disulphide oxidoreductase was specifically upregulated by exogenous Met. The pyridoxal-phosphate-dependent enzyme was upregulated in response to both Cys and Met treatments.

### 2.5. Cysteine and Methionine Supplements Resulted in Distinct Metabolomic Profiles

**Figure 5:**
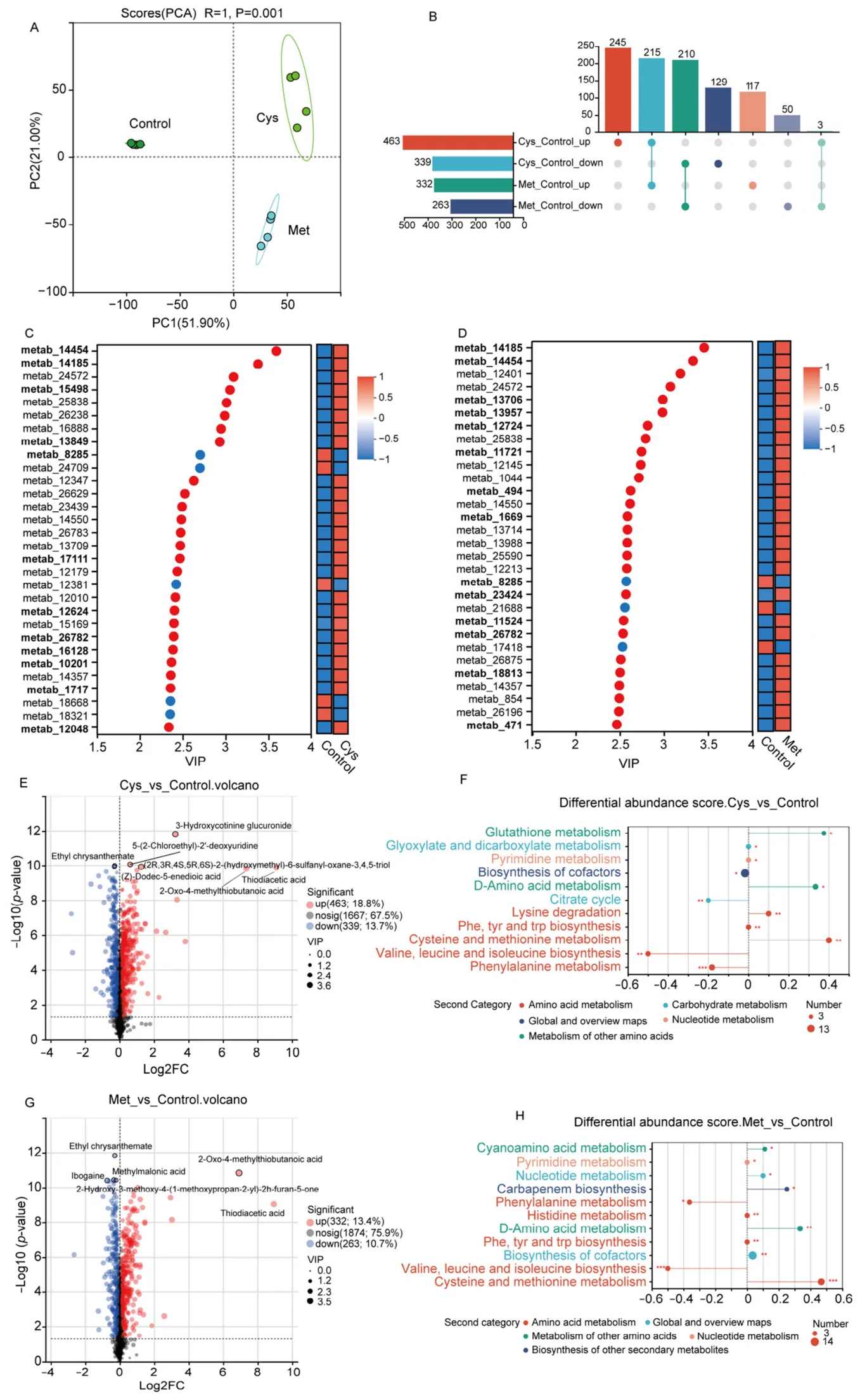
Metabolomic responses of *L. edodes* mycelium to exogenous cysteine (Cys) and methionine (Met). (**A**) Principal component analysis (PCA) of control and treated groups. (**B**) UpSet plot analysis showing differentially accumulated metabolites (DAMs) specific to Cys or Met treatment. (**C**,**D**) Top-ranked metabolites based on VIP scores derived from OPLS-DA models of cysteine supplementation versus control (**C**) and methionine supplementation versus control (**D**). The sulfur metabolites were highlighted in bold. (**E**,**F**) Volcano plot and KEGG pathway enrichment of DAMs in response to Cys supplementation. (**G**,**H**) Volcano plot and KEGG pathway enrichment of DAMs in response to Met supplementation. In volcano plots, point size reflects VIP values. In DA score analyses, point size represents the number of metabolites per pathway, and line length indicates the absolute DA score. Positive and negative DA scores correspond to pathway upregulation and downregulation, respectively. *, ** and *** indicate significant differences at *p* < 0.05, <0.01 and <0.001, respectively.

Non-targeted metabolomic analysis identified a total of 2471 metabolites, of which only 14.86 could be mapped to KEGG pathways (Supplementary Table S3). The annotations and expression levels of all identified metabolites are provided in Supplementary Table S4. Consistent with the proteomic results, metabolomic profiling revealed distinct metabolic responses of *L. edodes* mycelium to Cys and Met supplementation. PCA showed clear separation among treatments, with principal component 1 (PC1) and 2 (PC2) explaining 51.9% and 21.0% of the total variance, respectively. PC1 primarily distinguished samples with and without sulfur amino acid supplementation, whereas PC2 separated Cys- and Met-treated samples (Figure 5A). The UpSet plot identified 245 and 117 treatment-specific differentially accumulated metabolites (DAMs) in the Cys and Met groups, respectively (Figure 5B). The VIP values derived from OPLS-DA models were further used to rank metabolites according to their contribution to group discrimination. Metabolites with higher VIP values contributed more strongly to the separation between treatment and control groups. The abundance patterns and relative importance of individual DAMs are presented in the heatmap and VIP plots (Figure 5C,D). Among the top 30 DAMs ranked by VIP scores, 12 sulfur-containing metabolites were identified in the Cys group and 14 in the Met group, of which four were shared between treatments.

Volcano plot analysis identified 463 upregulated and 339 downregulated metabolites in response to Cys supplementation (Figure 5E). KEGG pathway enrichment analysis showed that these DAMs were predominantly associated with glutathione metabolism, D-amino acid metabolism, cysteine and methionine metabolism, and biosynthesis of cofactors (Figure 5F). In the Met-treated group, 332 metabolites were upregulated and 263 were downregulated relative to the control (Figure 5G). These DAMs were mainly enriched in cyanoamino acid metabolism, pyrimidine metabolism, and nucleotide metabolism. Similar to the Cys treatment, DAMs induced by Met were also significantly enriched in D-amino acid metabolism, cysteine and methionine metabolism, and biosynthesis of cofactors (Figure 5H).

### 2.6. Integrative Analysis Revealed Distinct “Sulfur Metabolite–Protein” Regulatory Networks Related to Exogenous Cysteine and Methionine Supplementation

**Figure 6:**
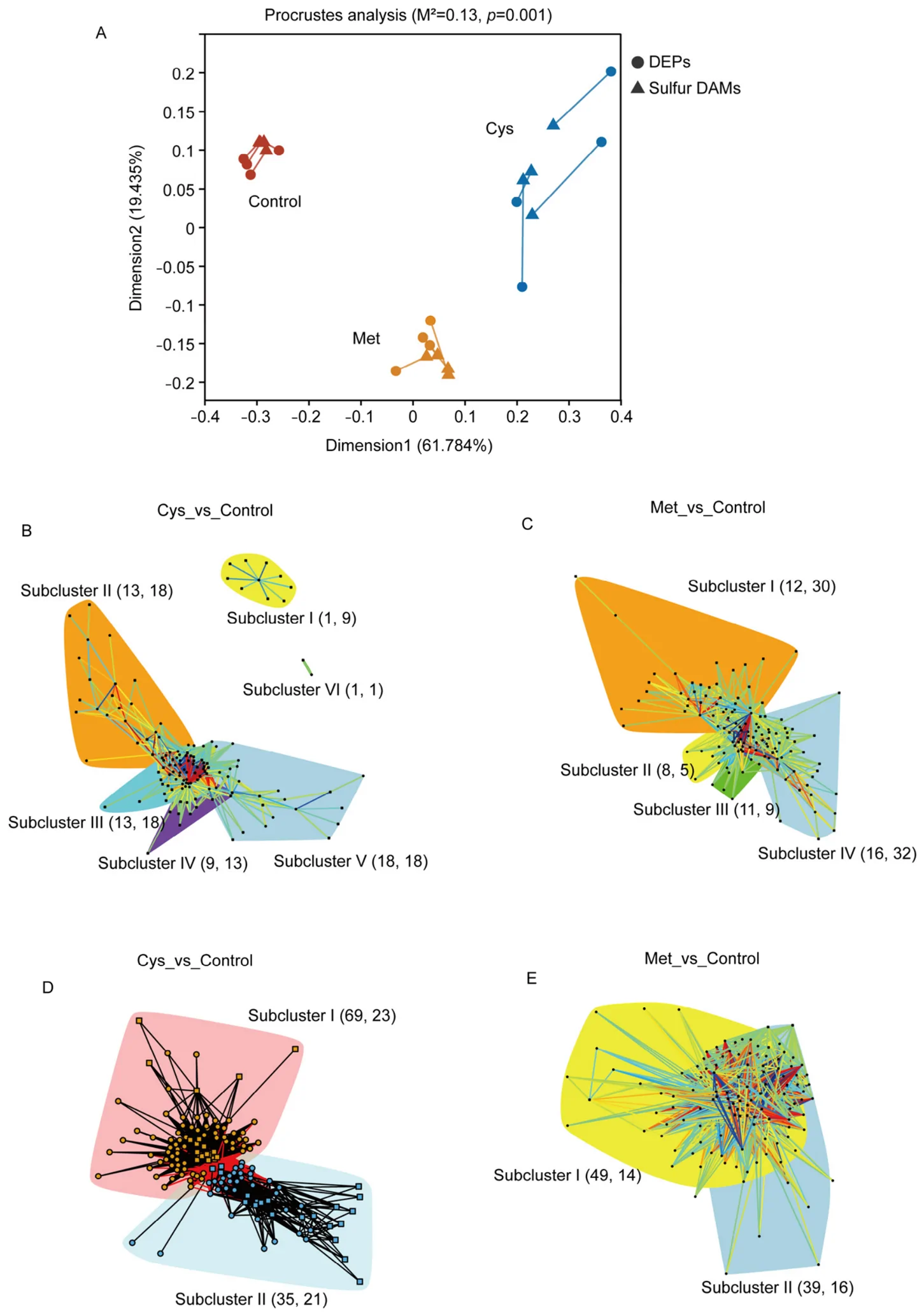
Integrative analysis of sulfur metabolites and proteins. (**A**) Procrustes analysis. (**B**,**C**) Network analysis of differentially accumulated sulfur metabolites (sulfur DAMs) and all differentially expressed proteins (DEPs). (**D**,**E**) Network analysis of sulfur DAM and DEP function in metabolism of sulfur metabolites. The value in brackets indicates number of sulfur metabolites and number of associated proteins.

To elucidate “metabolite–protein” regulatory networks, the sulfur DAMs were selected and used for integration analysis with DEPs. Firstly, we used Procrustes analysis to test the consistency between the sulfur DAM and the DEP datasets. The resulting arrow plot indicated a strong association between the two datasets in all treatment groups (Figure 6A). For Cys treatment, a total of six regulatory modules (subclusters I–VI) were identified, while four modules were found for Met treatment (Figure 6B,C). Further, we identified the DEP function in regulations of sulfur metabolites based on domain analysis, and performed an integrative analysis with sulfur DAMs to build a specific regulatory network between sulfur metabolites and proteins regulating sulfur metabolisms. Two regulatory modules were identified from datasets of exogenous Cys and Met, respectively (Figure 6D and Figure 6E). All the proteins and metabolites identified in each cluster are included in Supplementary Table S5. The top 10 hub proteins were identified, only 2 of which (p04275, p05403) were shared with both treatments (Table 1). For the hub proteins of Cys- and Met-derived networks, five of those are pyridine nucleotide-disulphide oxidoreductase which play a critical role in sulfur metabolism by catalyzing the transfer of reducing equivalents (electrons) to and from sulfur-containing compounds. The proteins with function of ATP-sulfurylase, nitrite and sulphite reductase 4Fe-4S domain were only present in the Cys-derived network, while the proteins with function of glutathione S-transferase, arylsulfotransferase were only present in the Met-derived networks (Table 1).

The transcriptional expression patterns of corresponding genes encoding the identified hub proteins were examined using qPCR (Supplementary Table S6 and Figure S2). Weak correlations were observed between qPCR-based transcription level analysis and sequencing-based relative expression levels of proteomic analysis.

## 3. Discussion

**Figure 7:**
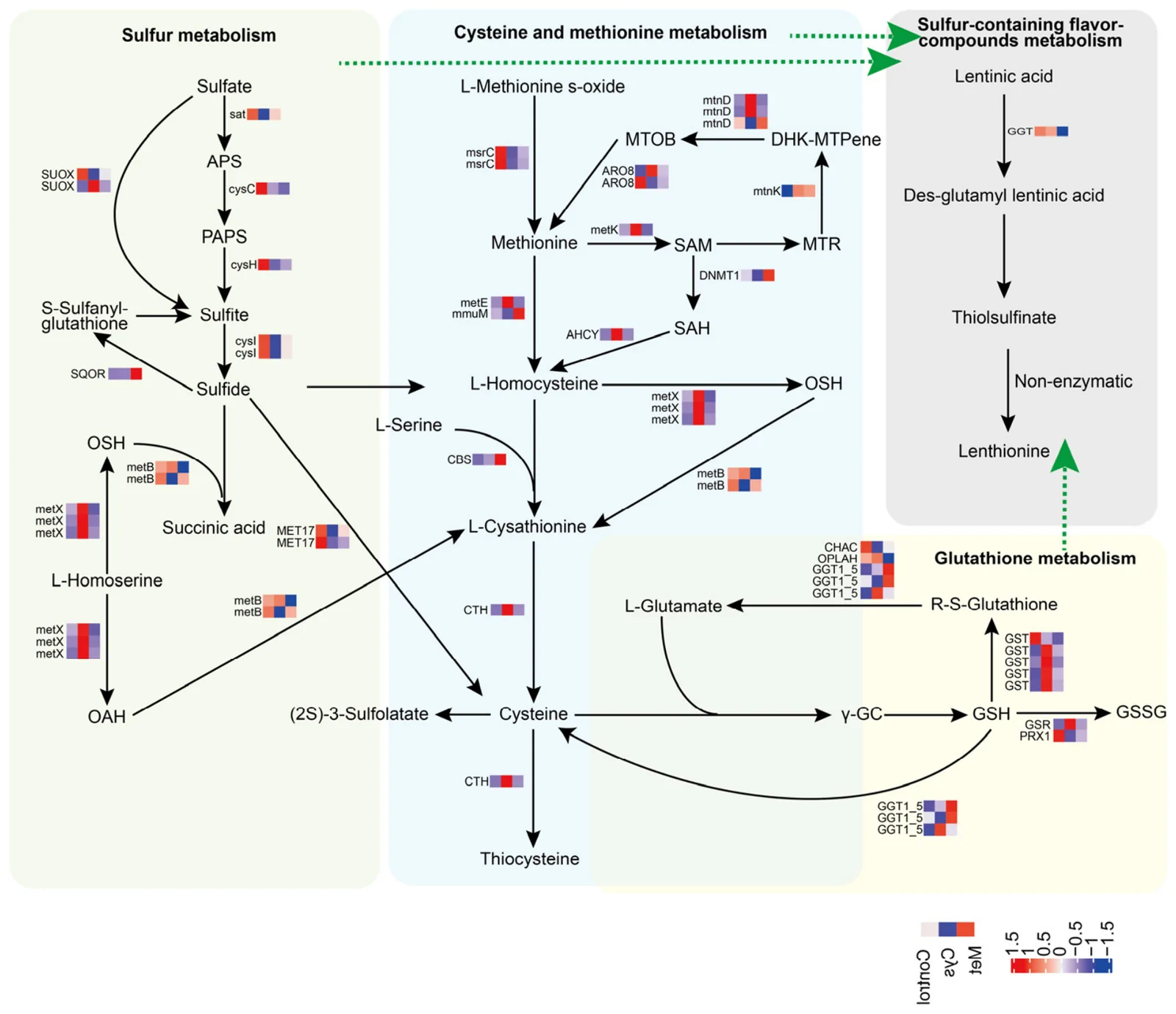
Schematic model illustrating the potential associations between sulfur-containing flavor metabolite (SCFM) biosynthesis and related metabolic pathways. The green dashed arrows indicate the possible involvement between SCFM biosynthesis and sulfur metabolism, cysteine and methionine metabolism, and glutathione metabolism. SAM, S-Adenosyl-L-methionine; SAH, S-Adenosyl-L-homocysteine; DHK-MTPene, 1,2-dihydroxy-3-keto-5-methylthiopentene; MTR, S-methyl-5-thio-D-ribose; MTOB, 4-methylthio-2-oxobutanoate; OSH, O-Succinyl-L-homoserine; γ-GC, Gamma-glutamylcysteine; OAH, O-Acetyl-L-homoserine; APS, Adenosine 5′-phosphosulfate; PAPS, 3′-Phosphoadenosine 5′-phosphosulfate; sat, sulfate adenylyltransferase; cysC, adenylylsulfate kinase; cysH, phosphoadenosine phosphosulfate reductase; cysI, sulfite reductase (NADPH) hemoprotein beta-component; SUOX, sulfite oxidase; SQOR, sulfide quinone oxidoreductase; metX, homoserine O-acetyltransferase; metB, cystathionine gamma-synthase; met17, O-acetylhomoserine/O-acetylserine sulfhydrylase; msrC, L-methionine (R)-S-oxide reductase; metK, S-adenosylmethionine synthetase; mtnD, 1,2-dihydroxy-3-keto-5-methylthiopentene dioxygenase; ARO8, aromatic amino acid aminotransferase I/2-aminoadipate transaminase; mtnK, 5-methylthioribose kinase; DNMT1, DNA (cytosine-5)-methyltransferase 1; AHCY, adenosylhomocysteinase; metE, 5-methyltetrahydropteroyltriglutamate–homocysteine methyltransferase; mmuM, homocysteine S-methyltransferase; CBS, cystathionine beta-synthase; CTH, cystathionine gamma-lyase; GGT, gamma-glutamyltranspeptidase; CHAC, glutathione-specific gamma-glutamylcyclotransferase; OPLAH, 5-oxoprolinase (ATP-hydrolysing); GST, glutathione S-transferase; GSR, glutathione reductase (NADPH); PRX1, glutaredoxin/glutathione-dependent peroxiredoxin.

The typical aroma of *L. edodes* is largely formed by sulfur-containing metabolites, forming the main part of its commercial asset. It is well established that primary sulfur metabolites are involved in the formation of the sulfur flavoring of shiitake mushroom. Cysteine and methionine are the only two sulfur-containing amino acids playing a crucial role in protein biosynthesis and located in the central position in the biosynthesis of various sulfur metabolites [19,20]. Those two amino acids have been shown to improve the formation of the sulfur flavor of shiitake mushroom fruiting bodies [17]. Further, cysteine was shown to enhance lenthionine biosynthesis under both normal and heat stress conditions [15]. The specific pathways and underlying molecular linkages are, however, still unclear. Here, using an integrative metabolomic and proteomic analysis, we demonstrated a conceptual framework of the pathways, functional proteins and sulfur intermediates that are potentially involved in the enhanced lenthionine biosynthesis by cysteine and methionine (Figure 7).

Primary sulfur metabolism comprises the assimilatory reduction of sulfate to sulfide, followed by its sequential incorporation into the sulfur-containing amino acids cysteine and homocysteine. It is worth noting that the expression level of the few functioning proteins in the pathway such as sat, cysC, cysH and cysl were decreased in response to exogenous cysteine and methionine. This could be explained by an adaptation response mediated by sulfur metabolite-dependent feedback regulation. Feedback regulation by downstream metabolites is a common mechanism for maintaining metabolic homeostasis [21]. For example, SAT, the limiting enzyme of cysteine biosynthesis, displays varying degrees of feedback inhibition by cysteine [22]. Similarly, glutamate-cysteine ligase, the rate-limiting enzyme of glutathione biosynthesis, is regulated by glutathione levels [23]. In fungi, sulfur metabolism is also controlled by sulfur metabolite repression, in which reduced sulfur compounds regulate the expression and abundance of proteins involved in sulfate uptake and assimilation. Early studies in *Neurospora crassa* demonstrated that methionine and cysteine availability regulates the synthesis and turnover of sulfur metabolic enzymes [24]. Recent study further confirmed that sulfur availability modulates fungal sulfur assimilation pathways through sulfur-responsive regulatory networks [25]. Therefore, the decreased abundance of sulfur assimilation-associated proteins observed in the present study may reflect reduced demand for de novo inorganic sulfur assimilation under conditions with sufficient exogenous organic sulfur sources, rather than decreased overall sulfur utilization.

More interestingly, the expression level of metK, which functions in the conversion of methionine to SAM, was downregulated by exogenous methionine. However, regulations of metK are sophisticated. In fungi, it is dynamically regulated in a multibranched biosynthetic pathway and multilevel, rather than solely depending on the substrate availability [26,27,28]. For example, in *Saccharopolyspora erythraea* metK transcription and SAM biosynthesis are regulated by multiple nutritional regulators, including the phosphate response factor PhoP and the nitrogen response factor GlnR [27]. In *Aspergillus nidulans*, SAM synthetase participates in the regulation of fungal development and secondary metabolism, indicating that MetK function extends beyond simple substrate conversion [26]. In *L. edodes*, however, the regulatory network of metK remains unclear. Furthermore, metK can be regulated by product availability, and metK inhibition by its product SAM has been reported from *Corynebacterium glutamicum* [29] and *Bacillus subtilis* [30]. In the present study, the SAM abundance was significantly elevated (1.5-fold) by exogenous methionine supplementation. Therefore, the decreased expression level of MetK observed following methionine supplementation may represent an adjustment to maintain SAM homeostasis in response to increased SAM availability rather than direct inhibition by methionine. The cysteine treatment, however, made no significant difference in SAM abundance. Moreover, cysteine does not directly regulate MetK in a demonstrated manner. Instead, cysteine can alter cellular sulfur homeostasis and redox status, which may indirectly affect SAM metabolism and SAM-dependent processes [25,26].

The normal media for *L. edodes* vegetative growth, for example, the potato dextrose agar (PDA) and MMN media, contain unknown background content of cysteine and methionine, influencing the accuracy of cysteine and methionine effect tests. Here, we used modified MMN media free of cysteine and methionine that enable more precise testing of the effect of cysteine and methionine. Using such media, we confirmed that both cysteine and methionine could enhance lenthionine biosynthesis, with which the methionine had the most significant effects. Meanwhile, for improvements of other sulfur-containing flavor metabolites, for example, 1,2,4-trithiolane, the methionine outperformed cysteine [17]. Cysteine and methionine were not the sole source of sulfur. The modified MMN medium used in this study retained basal sulfur-containing components, including MgSO_4_ and thiamine hydrochloride, to maintain essential sulfur metabolism during vegetative growth. These basic sulfur components were maintained at identical concentrations among all experimental groups, enabling the precise evaluation of responses to exogenous cysteine and methionine.

Except for the enhanced lenthionine biosynthesis, the exogenous cysteine and methionine also significantly altered the metabolic and proteomic profiles. The most pronounced alteration happened on the glycosphingolipid (GSL) biosynthesis pathway, which is interconnected with the metabolism of sulfur-containing metabolites, concerning the production and utilization of the universal sulfate donor PAPS (3′-phosphoadenosine-5′-phosphosulfate). PAPS is the central, universal sulfate donor used by all living cells to drive sulfation reactions. However, whether PAPS mediated the biosynthesis of sulfur-flavor metabolites remains to be elucidated. The upregulated GGT (rna_Led05741.1_sp3) which catalyzes the degradation of GSH to release cysteine, can also catalyze the critical first step of lenthionine production by cleaving its specific gamma-glutamyl peptide bond of lentinic acid [16,31,32]. The GGT can also transfer this gamma-glutamyl group to a water molecule or another amino acid acceptor, releasing free glutamate as a byproduct to contribute sulfur-flavor biosynthesis again [17,33]. In addition, in the glutathione metabolism pathway, 5 glutathione S-transferase (GST) proteins were significantly upregulated by Cys treatment as well. GST plays a crucial role in catalyzing the conjugation of glutathione and regulates intracellular pools of glutathione, cysteine, and sulfur-containing intermediates [34,35]. Based on current knowledge, GST is unlikely to be a direct enzyme that produces lenthionine. However, based on its established roles in glutathione metabolism, sulfur trafficking, redox regulation, and secondary metabolism [36,37], GST may function as an upstream regulator of sulfur-containing flavor biosynthesis in *L. edodes.* This regulatory role could be mediated through the regulation of glutathione/cysteine sulfur flux or maintenance of a favorable redox environment for sulfur metabolite formation. Except for GST and GGT, there are also several other identified DEPs function in the regulation of sulfur metabolite biosynthesis including Cys/Met metabolism PLP-dependent enzyme, S-adenosyl-L-homocysteine hydrolase, pyridine nucleotide-disulphide oxidoreductase and pyridoxal-phosphate dependent enzyme. Those enzymes are all crucial functional proteins in sulfur-amino acid metabolism and glutathione metabolism [38,39,40]. They may establish a functional network linking methionine cycling, cysteine biosynthesis, sulfur transfer, and redox regulation, thereby coordinating the production of sulfur-containing precursors and reactive intermediates required for the biosynthesis of lenthionine and other characteristic sulfur aroma compounds in *L. edodes*. In metabolomic analysis, S-adenosyl-L-methionine (SAM) and S-adenosyl-L-homocysteine (SAH), which serve as essential intermediate carriers for the conversion of methionine to cysteine, were significantly increased following cysteine and methionine supplementation. The accumulation of these metabolites suggests an altered methionine-associated metabolic state and may indicate enhanced activity of sulfur metabolism-related pathways. Additionally, the increased abundance of L-methionine S-oxide, 2-oxo-4-methylthiobutanoic acid, and homoserine suggests their potential involvement in methionine and cysteine metabolism and may contribute to maintaining sulfur-containing precursor availability. However, whether these metabolic changes represent increased metabolic flux or direct conversion between specific intermediates requires further validation using approaches such as isotope tracing and targeted enzymatic analyses.

The integrative analysis between the sulfur DAMs and DEPs responsible for sulfur DAM metabolism allows us to construct a regulatory network of “metabolites–proteins”. Exogenous cysteine and methionine resulted in distinct regulatory networks comprising different regulatory modules. Of those top 10 hub proteins, five are pyridine nucleotide-disulphide oxidoreductase and four are Cys/Met metabolism PLP-dependent enzyme, implying their role in modulation of sulfur-flavor metabolite biosynthesis. The qPCR analysis could provide complementary evidence for the transcriptional regulation of candidate hub proteins, but does not represent a direct replacement for protein-level validation. Weak correlations were observed between the qPCR-based transcription level analysis and sequencing-based relative expression levels of proteomic analysis. Such discrepancies can be attributed to a combination of post-transcriptional and post-translational regulatory mechanisms [41,42].

The typical aroma is usually characterized in fruiting bodies. However, the mycelial stage also represents an important model for investigating the metabolic basis of SCFM biosynthesis [13,15,43,44]. The sulfur-containing aroma compounds, including the key metabolite lenthionine, have been detected in *L. edodes* mycelia, suggesting that the precursor supply and core metabolic processes involved in SCFM biosynthesis are already established during vegetative growth. Also, the well-characterized SCFM regulatory pathway mediated by GGT and CSL is characterized in mycelium as well, suggesting the metabolic pathway is a conserved process rather than a fruiting-body-exclusive reaction. It should be noted that the “metabolite–protein” network established in this study was constructed based on the mycelial stage and therefore primarily represents the molecular landscape associated with vegetative growth. Previous studies have demonstrated that *L. edodes* undergoes extensive transcriptional and metabolic reprogramming during the transition from mycelium to fruiting body [45,46,47,48]. These developmental transitions are accompanied by changes in nutrient allocation, tissue differentiation, developmental signaling, and secondary metabolism, which may reshape the abundance, activity, and connectivity of sulfur metabolism-associated proteins [45,46]. Nevertheless, the hub proteins and metabolite-associated modules identified in the mycelial stage may represent conserved metabolic nodes that participate in sulfur flux regulation and provide potential targets for understanding sulfur-containing flavor biosynthesis during fruiting-body development. Future integrated transcriptomic, proteomic, and metabolomic comparisons between the mycelium and fruiting body will be essential to elucidate the dynamic evolution of these regulatory networks.

In conclusion, our results provide a comprehensive conceptual framework illustrating how cysteine and methionine mediate the sulfur-flavor metabolite biosynthesis. Furthermore, we identified potential candidate intermediate sulfur metabolites and the functional proteins involved in their production.

## 4. Materials and Methods

### 4.1. Fungal Culture and Treatment

The *L. edodes* strain 0912, a widely cultivated strain in China, was used in this study. The strain is maintained at the Edible Fungi Laboratory, Institute of Plant Protection, Beijing Academy of Agriculture and Forestry Sciences.

Mycelial plugs (1 cm in diameter) were excised from actively growing cultures using a sterile cork borer and inoculated onto Petri dishes (9 cm in diameter) containing 20 mL of modified Melin–Norkrans (MMN) medium. The medium consisted of 10 g glucose, 0.25 g (NH_4_)_2_HPO_4_, 0.5 g KH_2_PO_4_, 0.15 g MgSO_4_·7H_2_O, 0.05 g CaCl_2_·2H_2_O, 0.025 g NaCl, 15 g agar, 100 μL thiamin HCL (1 g/L), 1 mL FeCl_3_ (10 g/L), and 10 mL of microelement solution per 1 L of medium. The microelement solution comprised 3.728 g/L KCl, 1.546 g/L H_3_BO_3_, 0.845 g/L MnSO_4_·H_2_O, 0.575 g/L ZnSO_4_·7H_2_O, 0.125 g/L CuSO_4_·5H_2_O, and 0.048 g/L Na_2_MoO_4_. After autoclave, cysteine and methionine solutions were added to obtain a final concentration of 0.3, 0.6, and 0.9 g L^−1^ for cysteine, and 0.2, 0.4, and 0.6 g L^−1^ for methionine. Cysteine and methionine solutions were filtrated with a polyethersulfone (PES) sterile filter (pore size 0.22 μm, Whatman^®^, Cytiva, Maidstone, UK) to avoid contamination. Prior to inoculation, a sterile cellophane membrane was placed on the surface of the medium to facilitate subsequent mycelial harvesting [49]. The cultures were incubated at 25 °C in darkness for 7–9 days.

The phenotypic investigation was performed on Petri dish cultures. For enzymatic activity measurements and omics assays, liquid cultures were used because they allow more efficient pure biomass collection, and better homogeneity of samples. To obtain pure mycelial biomass, the mycelium cultured on Petri dishes were carefully collected and transferred to a liquid medium containing 2 g malt extract, 2 g glucose, and 200 mL deionized water. Liquid cultures were incubated at 25 °C with shaking at 160 rpm for 7–10 days. Following mycelial establishment, the cultures were divided into control and treatment groups. For the treatment groups, filter-sterilized cysteine (0.3, 0.6, and 0.9 g L^−1^) or methionine (0.2, 0.4, and 0.6 g L^−1^) was added aseptically to the culture medium at the designated concentrations. The cultures were subsequently incubated for an additional 12 h before sample collection. The 12 h sampling time point was selected based on preliminary time-course experiments examining the activities of key enzymes associated with SCFM biosynthesis, including GGT and CSL. These assays showed that both GGT and CSL activities reached their maximum levels approximately 12 h after cysteine supplementation (Supplementary Figure S3), suggesting that this time point represents an active response phase during cysteine-induced sulfur metabolism regulation.

### 4.2. Measurement of Average Colony Expansion Rate

The fungal average colony expansion rate was determined by measuring the average colony diameters of both the control and treatment groups on Petri dish cultures following the previously described method [50]. The growth rate was expressed as millimeters per day (mm d^−1^).

### 4.3. Lenthionine Content Determination

Fresh mycelial samples (0.1 g) from each treatment were mixed with 1 mL of acetonitrile and homogenized at 60 Hz for 10 min. Subsequently, 4 mL of acetonitrile was added, and the mixture was vortexed for 1 min. The samples were then sonicated in an ice-cold water bath (4 °C) for 30 min, followed by centrifugation at 13,000× *g* for 30 min. The extraction procedure was repeated once using an additional 5 mL of acetonitrile. The resulting supernatants were combined and evaporated to dryness under a gentle stream of nitrogen at room temperature. The dried residue was reconstituted in 1 mL of methanol, homogenized thoroughly, and subsequently diluted tenfold with methanol. The resulting solution was filtered through a 0.22 μm membrane filter prior to high-performance liquid chromatography (HPLC) analysis.

Lenthionine was quantified using an Agilent 1200 HPLC system (Agilent Technologies, Santa Clara, CA, USA) equipped with a variable-wavelength detector (VWD) and a C18 column (250 × 4.6 mm, 5 μm; Agilent Technologies, Santa Clara, CA, USA). The chromatographic conditions were as follows: column temperature, 35 °C; flow rate, 1.0 mL min^−1^; injection volume, 20 μL; and isocratic elution with methanol/water (90:10, *v*/*v*) as the mobile phase. Lenthionine was detected at a wavelength of 230 nm. Analytical grade lenthionine (purity ≥ 95%; Chembest, Shanghai, China) was used as an external standard. Calibration curves were established using standard solutions at concentrations of 0.05, 0.1, 0.2, 0.5, and 1.0 mg/L by plotting peak area against corresponding lenthionine concentration. The calibration curve exhibited excellent linearity with a coefficient of determination R^2^ of 0.9997 (Supplementary Figure S4). Representative chromatograms of lenthionine standards and biological samples are shown in Supplementary Figure S4. The limits of detection (LOD) and quantification (LOQ) were calculated using the standard residual error method and were determined to be 0.028 mg/L and 0.084 mg/L, respectively.

### 4.4. Measurements of GGT, CSL Enzymatic Activity and Intracellular Cysteine Content

Fresh mycelial samples (100 mg) were used to measure GGT and CSL activities using commercial assay kits (catalog number: BC1225 and BC5395, respectively; Beijing Solarbio Science & Technology, Beijing, China). For cysteine content determination, 200 mg of fresh mycelium was processed with a cysteine assay kit (catalog no. BC0185; Solarbio), following the manufacturer’s protocols. GGT and CSL activities were determined by measuring absorbance at 405 nm and 505 nm, respectively, while cysteine content was determined at 600 nm. All absorbance was obtained using a VersaMax microplate reader (Molecular Devices, San Jose, CA, USA).

### 4.5. Quantitative Real-Time PCR Analysis of Relative Expression Levels of ggt and csl

Total RNA was extracted using a TransZol Plant kit (TransGen Biotech, Beijing, China) according to the manufacturer’s instructions, and RNA quality was assessed using an Agilent 2100 Bioanalyzer (Agilent Technologies, Santa Clara, CA, USA). First-strand cDNA was synthesized from each sample using the TransScript Uni All-in-One First-Strand cDNA Synthesis SuperMix for qPCR (No. AT341, TransGen Biotech, Beijing, China). Quantitative real-time PCR (qPCR) was performed using a QuantStudio 7 Flex Real-Time PCR System (Thermo Fisher Scientific, Waltham, MA, USA) with PerfectStart Green qPCR SuperMix (No. AQ601, TransGen Biotech, Beijing, China). Relative gene expression levels were calculated using the 2^−ΔΔCT^ method [51]. The β-tubulin was used as reference to normalize the expression of examined genes. Primers used for qPCR analysis of *ggt* and *csl* are listed in Table 2. The melting curves revealed good specificities of those primers (Supplementary Figure S5). Primers used for the hub proteins are provided in Supplementary Table S6.

### 4.6. Non-Targeted Metabolomic Analysis

A total of 100 mg of mycelium was used for metabolite extraction. Each treatment is replicated 4 times. Samples were homogenized using a frozen tissue grinder for 6 min at −10 °C and 50 Hz, followed by ultrasonic extraction in an ice bath for 30 min at 40 kHz. The extracts were then incubated at −20 °C for 30 min and centrifuged at 13,000× *g* for 15 min at 4 °C. The resulting supernatant was transferred to an injection vial with an insert for UHPLC–MS/MS analysis. L-2-chlorophenylalanine (0.02 mg/mL) was used as the internal standard. As a part of the system conditioning and quality control process, a pooled quality (QC) control sample was prepared by mixing equal volumes of all samples. The QC samples were analyzed in the same manner as the analytic samples.

Metabolomic profiling was performed using an ultra-high-performance liquid chromatography (UHPLC) system (Thermo Fisher Scientific, San Jose, CA, USA) coupled with a Q Exactive mass spectrometer (Thermo Fisher Scientific, San Jose, CA, USA). A 3 μL aliquot of each sample was separated on an HSS T3 column (100 mm × 2.1 mm i.d., 1.8 μm) prior to mass spectrometric detection. The mobile phases consisted of 0.1% formic acid in water (solvent A) and 0.1% formic acid in acetonitrile (solvent B). All reagents were of MS grade. The flow rate was set at 0.40 mL/min, the column temperature was maintained at 40 °C, and the injection volume was 5 μL. The mass spectrometer was equipped with an electrospray ionization (ESI) source operating in both positive and negative ion modes. The optimized source parameters were as follows: source temperature, 400 °C; sheath gas flow rate, 40 arbitrary units (arb); auxiliary gas flow rate, 10 arbitrary units (arb); and ion spray voltage floating (ISVF), −2800 V in negative ion mode. Data were acquired in both positive and negative ion modes over a mass range of *m*/*z* 70–1050.

Raw LC–MS data were processed using Progenesis QI for proteomics v4.1 v4.1 (Waters Corporation, Milford, MA, USA) for baseline correction, peak detection, alignment, integration, and retention time correction. Mass identification was performed according to the established protocol [52]. The precursor ion mass tolerance was set to 10 ppm, and the product ion mass tolerance was set to 0.02 Da. For metabolites with MS/MS confirmation, only metabolites with MS/MS fragment scores > 30 were considered confidently identified. Metabolites showing full-spectrum matching with reference standards were assigned to identification level B(i). The metabolite MS/MS spectra matching against computer-simulated, theoretical spectra found in public or theoretical databases were assigned as level B(ii). The detailed information, including metabolite annotation, quantification, MS/MS fragmentation score, *m*/*z* value, identification level, and mass error, is provided in Supplementary Table S4. The resulting data matrix, including retention time, mass-to-charge ratio (*m*/*z*), and peak intensity, was annotated by matching against the Human Metabolome Database (HMDB, http://www.hmdb.ca/), the Metlin database (https://metlin.scripps.edu/) and the self-compiled Majorbio database (MJDB) (Majorbio Biotechnology, Shanghai, China) for metabolite identification.

Differentially accumulated metabolites (DAMs) between groups were identified using the criteria of variable importance in projection (VIP) ≥ 1 in the OPLS-DA model (Supplementary Figure S6), false discovery rate (FDR) ≤ 0.05, and fold change (FC) ≥ 1.2 or FC ≤ 0.83. Identified DAMs were further mapped to biochemical pathways through KEGG-based enrichment and pathway analysis (http://www.genome.jp/kegg/, accessed on 8 June 2026). Statistical enrichment of pathways was evaluated using Fisher’s exact test implemented in the Python package SciPy (https://docs.scipy.org/doc/scipy/, accessed on 5 June 2026).

### 4.7. Proteomic Analysis

Fresh mycelial samples (100 mg) were suspended in protein lysis buffer (8 M urea and 1% SDS) supplemented with protease inhibitors to prevent protein degradation. Each treatment was replicated 4 times. The samples were homogenized using a high-throughput tissue grinder for three cycles of 180 s each, followed by non-contact cryogenic ultrasonication for 30 min. After centrifugation at 16,000× *g* and 8 °C for 30 min, the supernatant was collected, and protein concentration was determined using a Bicinchoninic Acid (BCA) Protein Assay Kit (Thermo Fisher Scientific, Waltham, MA, USA) according to the manufacturer’s instructions. Protein quality was subsequently evaluated by SDS–PAGE.

For enzymatic digestion, 100 μg of protein was resuspended in triethylammonium bicarbonate (TEAB) buffer at a final concentration of 100 mM. Proteins were reduced with tris(2-carboxyethyl)phosphine (TCEP) at a final concentration of 10 mM and incubated at 37 °C for 60 min. Subsequently, proteins were alkylated with iodoacetamide (IAM) at a final concentration of 40 mM for 40 min at room temperature in the dark. Following centrifugation at 10,000× *g* and 4 °C for 20 min, the pellet was collected and resuspended in 100 μL of 100 mM TEAB. Trypsin was added at a trypsin-to-protein ratio of 1:50 (*w*/*w*), and digestion was carried out overnight at 37 °C. Following digestion, peptides were dried using a vacuum concentrator. The dried peptides were reconstituted in 0.1% trifluoroacetic acid (TFA), desalted using HLB cartridges, and subsequently dried again under vacuum. Peptide concentration was determined using a NanoDrop™ One Microvolume UV–Vis spectrophotometer (Thermo Scientific, Waltham, MA, USA) by measuring absorbance at 205 nm.

Based on peptide quantification results, samples were analyzed using a Vanquish Neo UHPLC system (Thermo Fisher Scientific, Waltham, MA, USA). Peptides were separated on a uPAC High Throughput column (75 μm × 5.5 cm; Thermo Fisher Scientific, USA) using solvent A (water containing 2% acetonitrile and 0.1% formic acid) and solvent B (80% acetonitrile containing 0.1% formic acid). The chromatographic run time was 8 min. Separated peptides were analyzed using an Orbitrap Astral mass spectrometer (Thermo Fisher Scientific, Waltham, MA, USA) coupled to the Vanquish Neo system. Data-independent acquisition (DIA) was performed with a mass scanning range of *m*/*z* 100–1700. DIA raw data were processed using Spectronaut software (Version 19). The database search parameters were as follows: peptide length, 7–52 amino acids; the minimum peptide length was 7 amino acids; digestion enzyme, trypsin/P; maximum missed cleavages, 2; fixed modification, carbamidomethylation of cysteine residues; and variable modifications, methionine oxidation and protein N-terminal acetylation. The false discovery rates (FDRs) for both proteins and peptides were controlled at ≤0.01. Peptide confidence was set to ≥99%, and the extracted ion chromatogram (XIC) width was set to ≤75 ppm. Protein quantification was performed using the MaxLFQ algorithm [53]. Missing values were defined as protein abundance values absent in ≥30% of samples within each experimental group and were subsequently imputed using the K-nearest neighbors (KNN) method [54]. The proteins that were absent in 70% of samples across every group simultaneously were removed. Peptide abundance data were normalized using local normalization method based on a retention time (RT)-dependent local regression model as described by Callister et al. [55]. Differentially expressed proteins (DEPs) between control and treatment groups were identified according to the criteria of fold change (FC) ≥ 1.2 or FC ≤ 0.83 and *p* ≤ 0.05. The *p* value and FC of proteins between control and treatment groups was calculated using the R package “*t*-test”.

Protein identification was performed against an integrated reference protein database generated from the proteins of the two haplotype genomes, SP3 and SP30. These two haplotypes represent sexually compatible monokaryotic strains derived from the *L. edodes* strain used in the present study [56]. The corresponding genome sequences of SP3 and SP30 are publicly available through the China National Center for Bioinformation (CNCB) under accession numbers GWHBGWX00000000 and GWHBGWY00000000, respectively. Functional annotation of all identified proteins was performed using GO (http://geneontology.org/, accessed on 5 June 2026) and KEGG pathway (http://www.genome.jp/kegg/, accessed on 5 June 2026). DEPs were further used for GO and KEGG pathway enrichment analysis. Protein–protein interaction analysis was performed using the String v11.5 [57].

### 4.8. Statistical Analysis

Principal component analysis (PCA) was performed to examine the overall variation and group separation of the proteomic and metabolomic data. Identification of DEPs and DAMs, PCA, and GO and KEGG enrichment analysis were conducted using the Majorbio Cloud platform (https://cloud.majorbio.com) [58]. Statistical significance between the control and treatment groups was evaluated using Student’s *t*-test. To elucidate the regulatory networks underlying sulfur-containing flavor biosynthesis in *L. edodes*, an integrated multi-omics analysis was conducted using sulfur metabolites with all differentially expressed proteins (DEPs) and also DEP function in metabolism of sulfur metabolites. The analytical workflow was established according to previously described methods [50,59]. Procrustes analysis was performed using the R package vegan (version 2.7-0) [60] to evaluate the overall concordance between the metabolomic and proteomic datasets. Prior to integration, metabolite and protein abundance matrices were scaled and centered to a mean of 0 and a standard deviation of 1 to remove scale bias. The protein ID prefixes “rna_Led”, “rna_Add” and “rna_New” were shortened as “*p*” to enable clearer visualization. Sparse partial least squares (sPLS) regression mode was performed using R package ‘mixOmics’ [61,62] to identify metabolite–protein associations. The sPLS model was used to select correlated metabolite and protein variables, and strong significant associations were retained based on an association threshold of ≥0.95. The regulatory community was detected using the leading eigenvector clustering algorithm implemented using the R package “igraph” (version 2.3.3.9017) [63]. The detected network was visualized using the R package “igraph” (version 2.3.3.9017) using the Fruchterman–Reingold layout algorithm [64]. The hub proteins were identified and ranked by calculating the node importance using the cytoHubba plugin [65] in Cytoscape (Version 3.10.4) [66]. The maximal clique centrality (MCC) algorithm was applied to evaluate the topological importance of each protein node within the metabolite–protein interaction network. The MCC score represents the number and size of maximal cliques associated with a given node, reflecting the degree of connectivity of that node within highly interconnected network modules. Proteins with higher MCC scores were considered topologically more central and were selected as candidate hub proteins.

## 5. Conclusions

This study demonstrated that exogenous cysteine and methionine supplementation significantly promoted lenthionine accumulation in *L. edodes* and induced extensive changes in both the proteome and metabolome in mycelium. Integrated proteomic and metabolomic analyses revealed that cysteine and methionine regulate sulfur-containing flavor metabolite biosynthesis through distinct metabolic pathways and metabolite–protein regulatory networks. Furthermore, several candidate hub proteins and intermediate metabolites potentially involved in sulfur-containing flavor biosynthesis were identified. These findings provide an integrated molecular framework linking SCFM biosynthesis with primary metabolic pathways and associated sulfur intermediates, offering new insights into the potential regulatory networks involved in SCFM formation in *L. edodes*.

## Figures and Tables

**Table 1:** Hub proteins putatively related to regulation of sulfur-containing metabolites.

	Rank	Protein ID	Score	*p* Value	Annotation
Cys Network	1	p05546	89	1.09 × 10^−7^	ATP-sulfurylase
	1	p09176	89	1.28 × 10^−7^	Nitrite and sulphite reductase 4Fe-4S domain
	3	p04275	88	3.89 × 10^−8^	Pyridine nucleotide-disulphide oxidoreductase
	4	p00395	87	8.33 × 10^−9^	Cys/Met metabolism PLP-dependent enzyme
	5	p03712	85	5.66 × 10^−7^	Cys/Met metabolism PLP-dependent enzyme
	6	p05403	84	2.39 × 10^−6^	Papain family cysteine protease
	7	p08432	82	2.83 × 10^−6^	Phosphoadenosine phosphosulfate reductase family
	8	p03688	81	2.88 × 10^−6^	Pyridine nucleotide-disulphide oxidoreductase
	8	p09331	81	2.31 × 10^−6^	Pyridine nucleotide-disulphide oxidoreductase
	10	p06304	78	4.72 × 10^−6^	O-methyltransferase
Met Network	1	p08994	81	1.04 × 10^−6^	Pyridoxal-phosphate dependent enzyme
	2	p00403	80	3.73 × 10^−6^	O-methyltransferase domain
	3	p01302	79	3.86 × 10^−1^	Pyridine nucleotide-disulphide oxidoreductase
	3	p06546	79	7.23 × 10^−6^	Glutathione S-transferase
	5	p00395	78	2.28 × 10^−7^	Cys/Met metabolism PLP-dependent enzyme
	5	p04275	78	1.45 × 10^−7^	Pyridine nucleotide-disulphide oxidoreductase
	7	p05403	77	5.15 × 10^−6^	Papain family cysteine protease
	8	p06586	76	3.10 × 10^−8^	Arylsulfotransferase
	8	p09792	76	6.16 × 10^−7^	Cys/Met metabolism PLP-dependent enzyme
	8	p03748	76	2.52 × 10^−6^	Glutathione S-transferase, N-terminal domain

Score: The MCC score represents the node importance calculated by the maximal clique centrality (MCC) algorithm, reflecting the number and size of maximal cliques associated with a given node. *p* value: Statistical significance level calculated between each treatment group and the control group.

**Table 2:** *ggt* and *csl* primer sequence.

Gene Name	Primer Name	Primer Sequence (5′→3′)
*csl1*	Csl-1-F	ACGAGATCGTCATGACCGCTAA
	Csl-1-R	GTACCATAGGTGGTGTTGAAGCG
*csl2*	Csl-2-F	TGGCCAGCACAACATTCCATACA
	Csl-2-R	ATGCAGCTGTGAAAGCTTTCCAA
*csl3*	Csl-3-F	GCTTGCTGAACGCTCAGGTC
	Csl-3-R	CCTGGAGAAGACGTGATACTCTCG
rna-Led09828.2-sp30 (*ggt1*)	rna-Led09828.2-sp30-F	AGCAACATCGTCGTAGCGG
	rna-Led09828.2-sp30-R	GCACAATGTCGGATGAGGG
rna-Led01546.1-sp30 (*ggt2*)	rna-Led01546.1-sp30-F	GGTTAGACGCCCAGTTGTCC
	rna-Led01546.1-sp30-R	CGCATCTGCCTGATCCTGT
rna-Led01547.2-sp30 (*ggt3*)	rna-Led01547.2-sp30-F	CCTCGTCACAACCATTAGCG
	rna-Led01547.2-sp30-R	AGTCACATTCAAGGCAGCAGA

## Data Availability

The raw proteomic and metabolomic data generated in this study are publicly available in the National Genomics Data Center (NGDC) under project accession number PRJCA067510, including OMIX018069 (metabolomic data) and OMIX018070 (proteomic data).

## Supplementary Materials

The following supporting information can be downloaded at: https://www.mdpi.com/article/10.3390/ijms27177744/s1.

## Abbreviations

The following abbreviations are used in this manuscript:

SCFMs	Sulfur-containing flavor metabolites
GGT	Gamma-glutamyl transferase
C-S lyase	Cysteine sulfoxide lyase
Cys	Cysteine
Met	Methionine
SAM	S-Adenosyl-L-methionine
SAH	S-Adenosyl-L-homocysteine
DHK-MTPene	1,2-Dihydroxy-3-keto-5-methylthiopentene
MTR	S-Methyl-5-thio-D-ribose
MTOB	4-Methylthio-2-oxobutanoate
OSH	O-Succinyl-L-homoserine
γ-GC	Gamma-glutamylcysteine
OAH	O-Acetyl-L-homoserine
VIP	Variable importance in projection
FC	Fold changes
FW	Fresh weight
