# Supplementary material for: Multi-Omics Analysis Reveals Molecular Networks and Key Pathways Associated with Cysteine- and Methionine-Mediated Biosynthesis of Sulfur-Containing Flavor Metabolites in Lentinula edodes
Source: Int J Mol Sci. 2026 Aug 29;27(17):7744. doi: 10.3390/ijms27177744 (PMC13565951; doi:10.3390/ijms27177744)
Supplement: Supplementary file 1 [file ijms-27-07744-s001.zip › Supplementary Figures-revised.pdf]

Supplementary figures

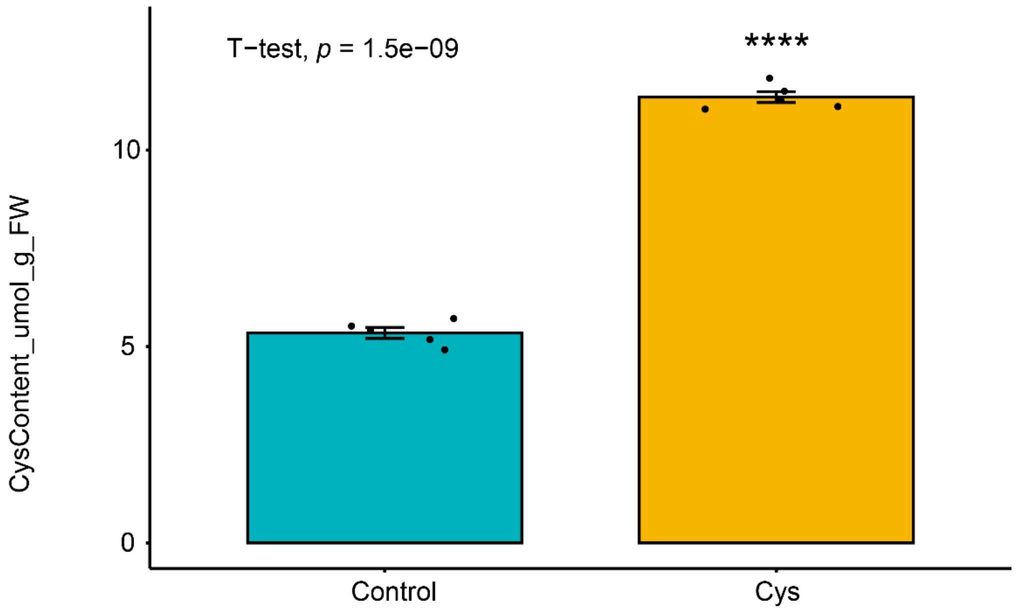

Supplementary Figure S1. Effects of exogenous cysteine on the intracellular cysteine content of *L. edodes* mycelia. Data were presented as mean ± SEM (n = 5 independent biological replicates). Error bars represent the standard error of the mean (SEM). **** indicate significant difference at $p < 0.0001$.

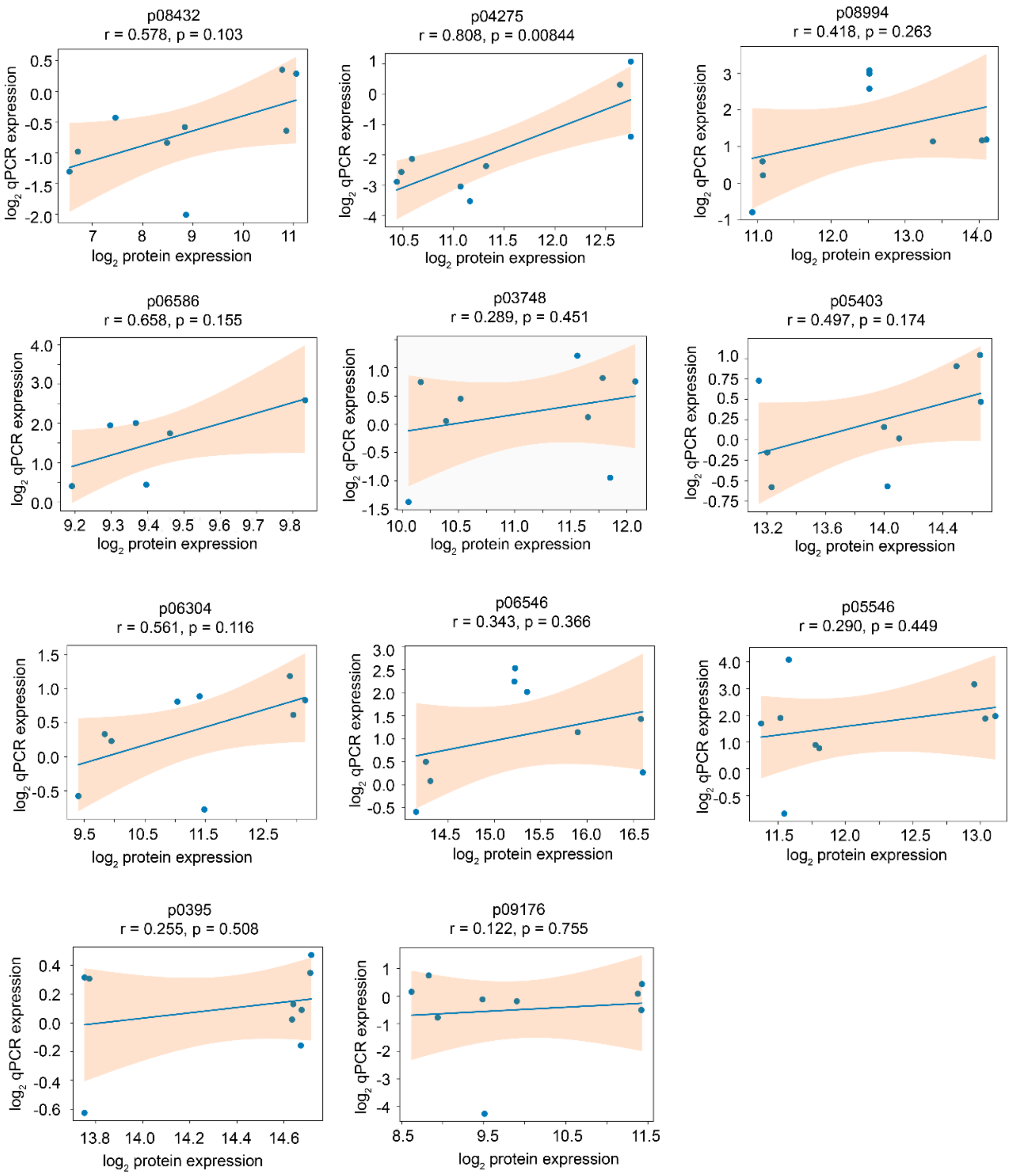

Supplementary Figure S2. Consistency analysis between the qPCR relative transcriptional levels and the proteomic data using Pearson correlation analysis. The scatter plot displays individual data points, with the blue line representing the linear regression fit and the brown shaded area indicating the 95% confidence intervals of the regression. The Pearson correlation coefficient (r) and corresponding *p* value indicate the strength and direction of the linear relationship, and statistical significance, respectively.

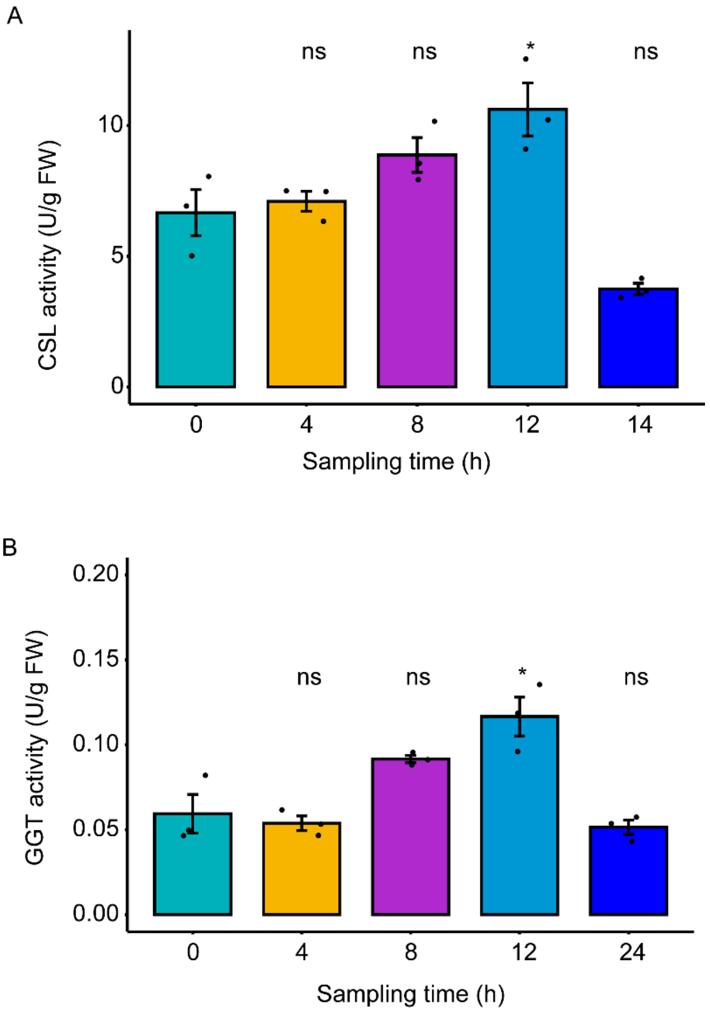

Supplementary Figure S3. Time-series analysis of CSL (A) and GGT (B) activities in response to cysteine supplementation. Data are presented as mean ± SEM (n = 4 biological replicates); error bars represent the standard error of the mean (SEM). The sampling time point 0 was used as the control to evaluate dynamic changes in CSL and GGT activities. * Indicates a significant difference at $p < 0.05$; "ns" indicates no significant difference compared to control. FW, fresh weight.

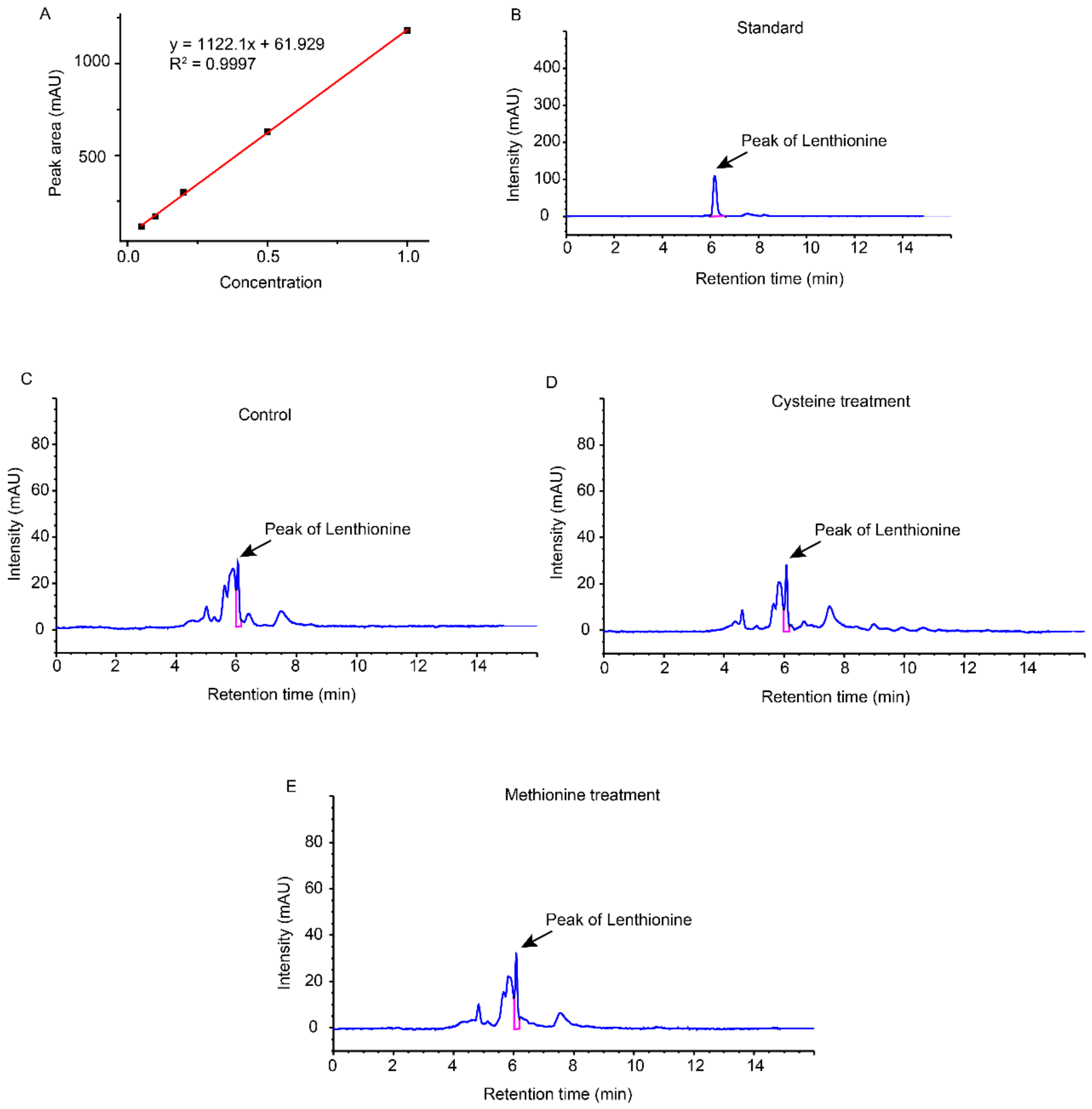

Supplementary Figure S4. The lenthionine measurements using HPLC. (A) calibration curve using standard; (B) HPLC peaks of lenthionine standard; (C–E) representative chromatograms of lenthionine in samples of control, cysteine and methionine treatments, respectively.

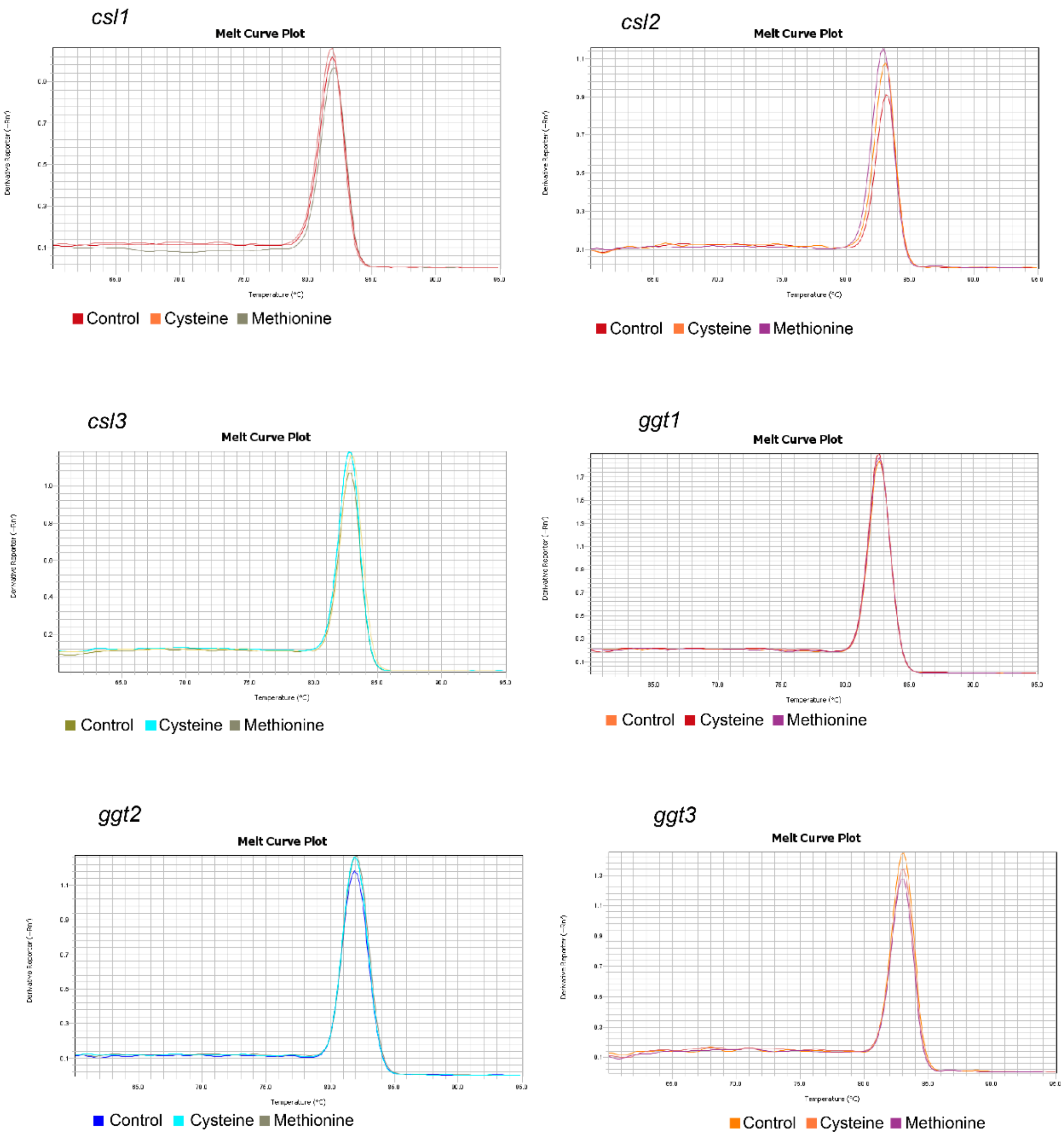

Supplementary Figure S5. The melting curves of examined genes in different treatments.

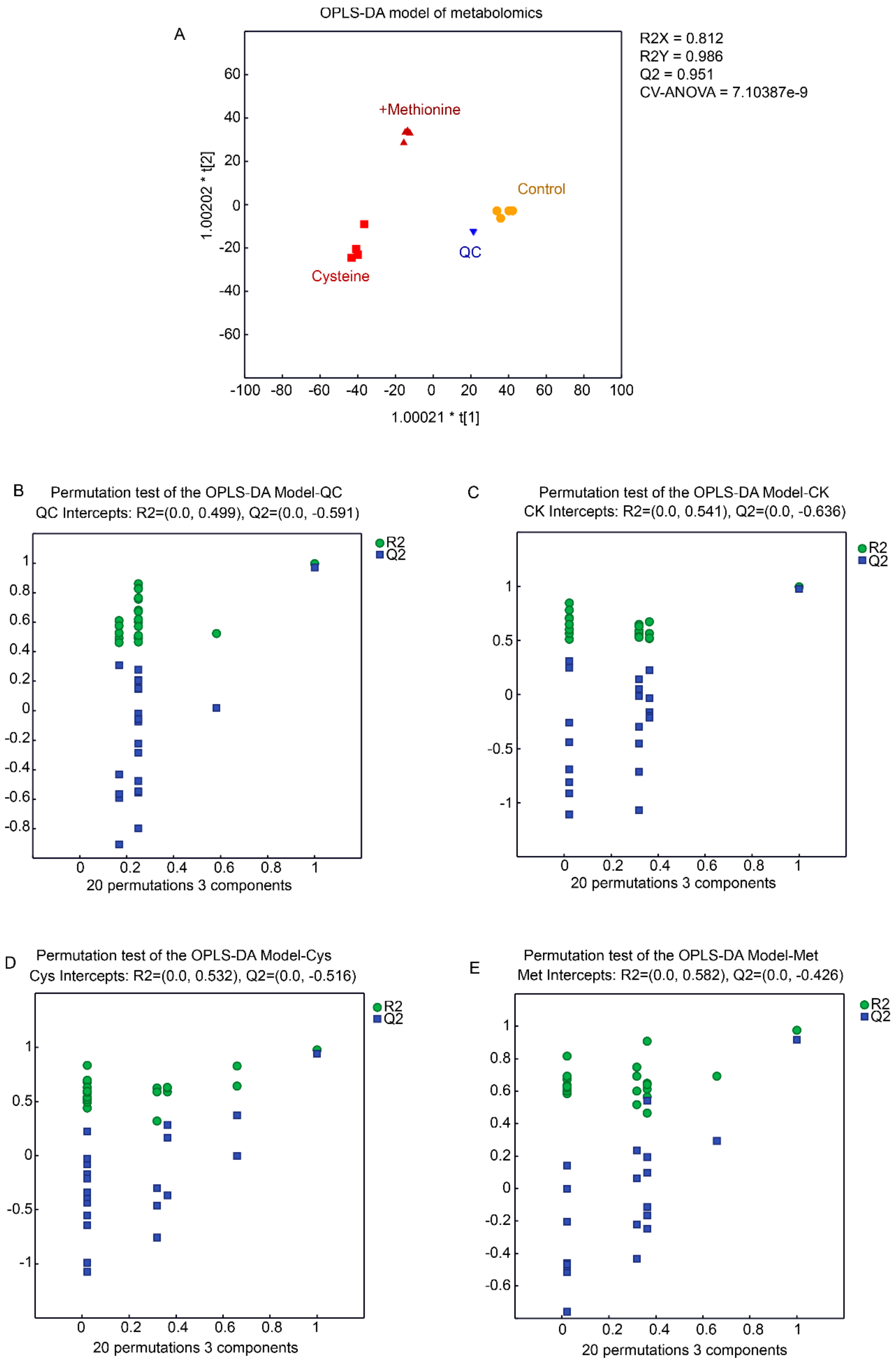

Supplementary Figure S6. OPLS-DA models for metabolomic datasets derived from exogenous cysteine and methionine supplementation. (A) Score plots of the OPLS-DA model; (B–E) permutation tests of the quality control (QC), control,

cysteine and methionine treatments, respectively. QC samples were prepared by combining equal volumes of all samples; Control group represents samples without exogenous amino acids supplementation.
